# Effective inhibition of cancer cells by recombinant adenovirus expressing EGFR-targeting artificial microRNA and reversed-caspase-3

**DOI:** 10.1371/journal.pone.0237098

**Published:** 2020-08-03

**Authors:** Maoxiao Yan, Jia Chen, Hua Jiang, Yuqiong Xie, Chunchun Li, Lihong Chen, Beibei Yang, Jiang Cao

**Affiliations:** 1 Department of Otorhinolaryngology, The Second Affiliated Hospital, School of Medicine, Zhejiang University, Hangzhou, Zhejiang Province, People’s Republic of China; 2 Clinical Research Center, The Second Affiliated Hospital, School of Medicine, Zhejiang University, Hangzhou, Zhejiang Province, People’s Republic of China; Sechenov First Medical University, RUSSIAN FEDERATION

## Abstract

The EGFR-targeting cancer therapies are commonly facing drug resistance, mostly due to mutations. Gene therapy with artificial microRNA targeting EGFR conserved sequence may avoid such problem. In this study, we constructed a recombinant adenovirus expressing EGFR-targeting artificial microRNA and active revCASP3 (Ad-EC), under the control of tumor-specific SLPI promoter, and evaluated its inhibitory effect on HEP-2 cancer cells both *in vitro* and *in vivo*. MTT assay showed that cell growth inhibition rate at 72h was 44.0% in Ad-EC group at MOI 50, while the rate was 7.7% in the control virus Ad-GFP group and 3.6% in Cetuximab (500 μg/ml) group respectively. Flow cytometry analysis revealed the late apoptotic cells rate was 36.1% in Ad-EC group, significantly higher than 6.5% of Ad-GFP group (*p* < 0.001). When Ad-EC (MOI 50) was combined with CDDP (0.25 μg/ml), late apoptotic cells rate increased to 61.2%, significantly higher than each monotherapy group (*P <* 0.001). The real-time xCELLigence system recorded an effective cell growth inhibition in Ad-EC and CDDP groups, and more enhanced effect in Ad-EC plus CDDP group. Western blot revealed that Ad-EC could inhibit the activation of AKT pathway and ERK1/2 pathway, while Cetuximab had the AKT pathway over-activated. *In vivo* experiments with HEP-2 xenograft in nude mice confirmed the tumor inhibition in Ad-EC, CDDP and Ad-EC plus CDDP groups compared with PBS group (*P <* 0.01). Collectively, these data support the effective inhibition of cancer cells by this novel gene therapy strategy.

## Introduction

Cancer is one of the major threats to human life. Advanced cancers are often refractory to surgical resection, for which radiotherapy/chemotherapy/biological therapy are needed. At present, the poor outcomes of survival rate and life quality are still the main problems in the treatment of advanced cancers. It is of clinical importance to explore new treatment with higher-efficiency and lower-toxicity.

Epidermal growth factor receptor (EGFR) overexpression/mutation is a common feature of many types of cancer including non-small cell lung cancer (NSCLC), gastrointestinal cancer, esophageal cancer, pancreatic cancer, head and neck squamous cell carcinoma (HNSCC), cervical cancer, *etc*., and contributes to malignant proliferation/metastasis [1–4]. EGFR-targeting therapy have been approved for the treatment of cancer with EGFR-overexpression/mutation. For instance, Osimertinib, a third-generation EGFR tyrosine kinase inhibitor (TKI) has been approved by the U.S. Food and Drug Administration for treatment of metastatic NSCLC patients with EGFR T790M mutation [4] which accounts for acquired resistance in 50%–60% of the patients who received first- and second-generation EGFR TKIs treatment [3, 5]. But more EGFR mutations keep arising [3]. Cetuximab, a monoclonal antibody was approved for metastatic colorectal cancers and HNSCCs, but also have a high resistance rate and common side effects to skin and gastrointestine [6, 7]. Many mechanisms for TKIs/monoclonal antibodies resistance have been reported such as bypass track signaling pathways, EGFR overexpression, degradation disorders, histologic transformation, oncogenic shift, target mutations, *etc*. [8, 9].

We previously designed an artificial microRNA targeting EGFR (EGFRamiR) to block the EGFR pathway at transcriptional level, which may avoid the above-mentioned resistance mechanisms of TKIs or monoclonal antibodies, like EGFR overexpression, mutation and degradation disorders. In previous studies, we confirmed that this microRNA effectively inhibited tumor cells *in vitro* [10, 11].

Caspase-3 acts as an executioner in apoptosis signaling. The reversed-caspase-3 (revCASP3) is artificially recombined from human natural caspase-3 which can spontaneously fold into an active state without cleavage by upstream initiator caspases and directly induce apoptosis [12, 13]. Our previous studies have demonstrated that forced expression of revCASP3 is effective in inducing tumor cell apoptosis [14].

SLPI is overexpressed in many types of cancer, such as ovarian cancer, breast cancer, and HNSCCs [15–18]. The promoter of SLPI has been introduced as a tumor-specific promoter to achieve specific expression of gene of interest in cancer gene therapy [14, 19]. In our previous study, we have confirmed that SLPI is highly expressed in HEP-2 cells (now denoted as HeLa contaminant by American Type Culture Collection), and recombinant adenovirus armed with revCASP3 under the control of SLPI promoter has specific tumor targeting [14]. In this work, a recombinant adenovirus carrying EGFR-targeted artificial microRNA and recombinant activated caspase-3 under the control of SLPI promoter was constructed. Using first-line drugs cisplatin (CDDP) and Cetuximab in control groups, the anti-cancer effect of the new gene therapy strategy which combines EGFR-inhibition and apoptosis-inducing was investigated on EGFR-overexpressing cancer cell HEP-2. HEP-2 is an ideal model for this study as it is resistant to erlotinib, a first generation of EGFR TKIs, and Cetuximab *in vitro* [20].

## Materials and methods

### Cell lines and animals

HEP-2 and HEK293 cell lines were purchased from Shanghai Institute of Biochemistry and Cell Biology, Chinese Academy of Sciences, and were both authenticated by STR profiling. Human normal fibroblast (NF) was a gift from Dr. Qing Yu. HEP-2 cells were maintained in Roswell Park Memorial Institute (RPMI) -1640 medium with 10% fetal bovine serum (FBS). HEK293 and NF cells were maintained in Dulbecco’s modified Eagle’s medium (DMEM) supplemented with 10% FBS. All cells were cultured at 37°C, 5% CO2 and 100% humidity, and were split when confluent. Thirty BALB/c-nu/nu male mice, 5–6 weeks old and 18–20 grams in weight, were obtained from Shanghai SLAC Laboratory Animal Co., Ltd (Shanghai, China). They were bred under the specific pathogen free environment and kept at a constant humidity (50–80%) and temperature (18–22°C) according to standard guidelines. The cage equipment, bedding, drinking water and feed were disinfected and sterilized.

### Generation of recombinant adenovirus Ad-EC

**Fig 1:**
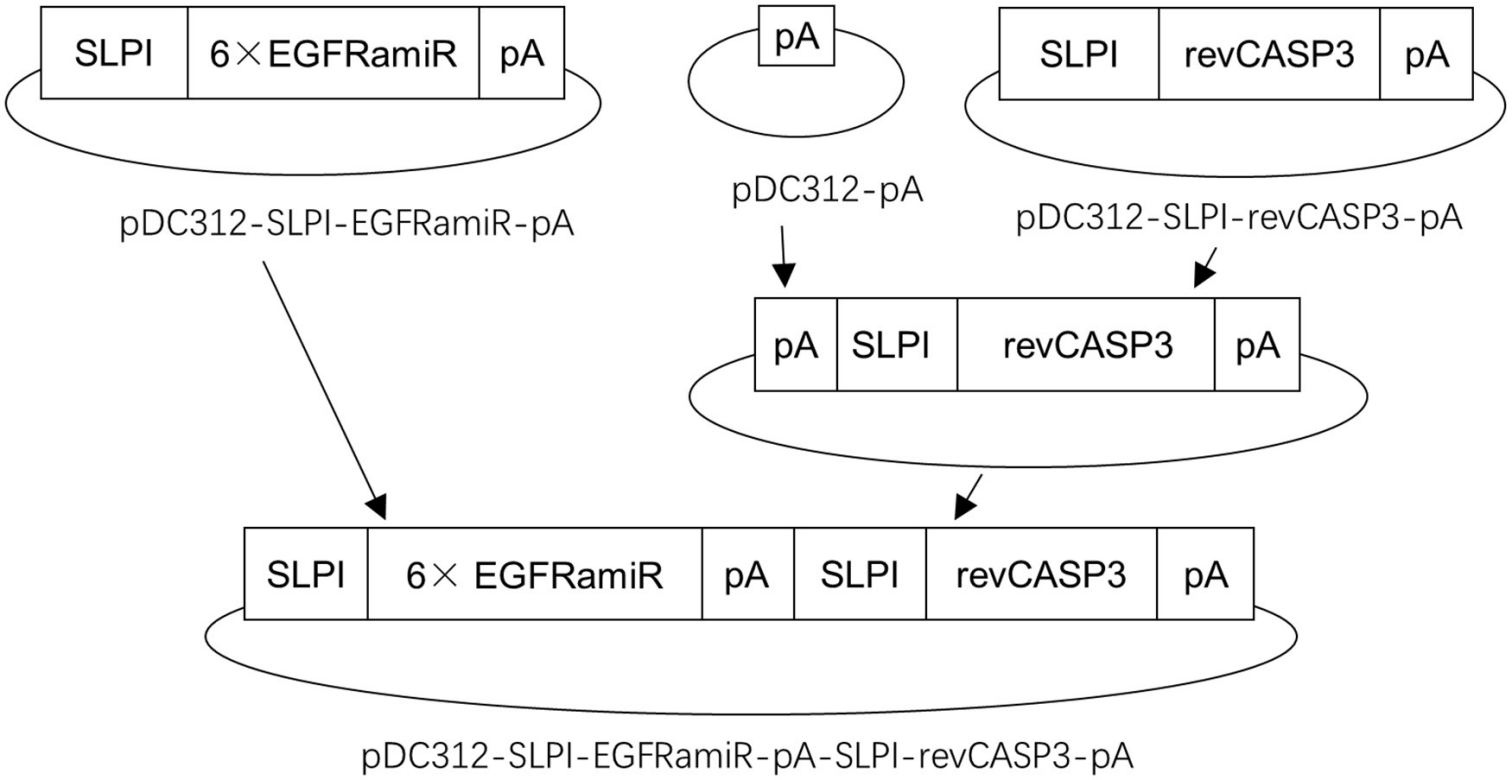
Strategy for pDC312-SLPI-EGFRamiR-pA-SLPI-revCASP3-TAG-pA construction.

The shuttle plasmid pDC312-SLPI-EGFRamiR-pA-SLPI-revCASP3-TAG-pA was constructed by joining two independent expression cassettes for EGFRamiR [10, 11] and revCASP3 [14] respectively, each controlled by a SLPI promoter for transcription initiation and a bovine growth hormone polyadenylation signal for transcription termination and mRNA polyadenylation. The strategy for plasmid construction was illustrated in Fig 1.

The AdMax^**™**^ System adenovirus packaging system (Microbix Biosystems, Toronto, Canada) was used for virus packaging, by which the shuttle plasmids with exogenous gene and adenovirus backbone plasmids with adenoviral genomic DNA were co-transfected into HEK293 cells, generating replication deficient adenoviruses after site-specific recombination between shuttle plasmids and backbone plasmids and packaging. The experimental procedure followed the instruction manual of the packaging system.

### Detection of the expression of targeted gene and the downstream signaling pathways

Fluorescence microscopy (Olympus, Tokyo, Japan) was used to observe the expression of GFP in HEP-2 cells and NF cells 48 h after Ad-GFP infection at a multiplicity of infection (MOI) of 50 pfu/cell. Western blotting was performed to examine the expression of proteins in HEP-2 cells, 72 h after the following treatments: Ad-EC (MOI 50), CDDP (0.25μg/ml), Ad-EC (MOI 50) combined with CDDP (0.25μg/ml), Cetuximab (500μg/ml), Ad-GFP (MOI 50) or PBS. CDDP (Nuoxin^®^, lot number: 140703) was the product of Jiangsu Hansoh Pharmaceutical Co., Ltd. (Jiangsu, China). Cetuximab (Erbitux^®^, lot number: 171709) was the product of Merck Pharma GmbH (Darmstadt, Germany).

Briefly, cells from six-well flat-bottom plates were harvested and lysed with RIPA buffer containing protease inhibitors (Sigma Aldrich, St. Louis, MO, USA). The proteins were separated by sodium dodecyl sulfate polyacrylamide gel electrophoresis and transferred to polyvinylidene fluoride membrane (Millipore, Bedford, MA, USA). The membrane was blocked with 5% nonfat milk in Tris-buffered saline containing 0.5% Tween-20 (TBS-T) for 1 h at room temperature, then incubated with primary antibodies against EGFR (ab32077; Abcam, Cambridge, MA, USA), caspase-3 (8G10; Cell Signaling Technology, Beverly, MA, USA), ADP-ribose polymerase (PARP)(46D11; Cell Signaling Technology), Protein kinase B (AKT) (9272; Cell Signaling Technology), phosphorylated AKT (9271; Cell Signaling Technology), extracellular signal-regulated kinase (ERK) (4696; Cell Signaling Technology), phosphorylated ERK (4370; Cell Signaling Technology), or GAPDH (KC5G5; KangChen Bio-tech, Shanghai, China) in TBS-T containing 5% bovine serum albumin at 4 °C overnight. After washing, the membranes were incubated with the enzyme horseradish peroxidase (HRP) -conjugated secondary antibodies for 2 h at room temperature. The specific bands were detected by Clarity^™^ Western ECL Substrate (Bio-Rad Laboratories, Hercules, CA, USA) and ChemiDoc MP Imaging System (Bio-Rad Laboratories).

### Measurement of growth inhibition

The growth inhibition effect of recombinant adenoviruses on HEP-2 cell line was examined by 3-(4,5-dimethylthiazol-2-yl)-2,5-diphenyltetrazolium bromide (MTT) assay. Briefly, 2 × 10^3^ cells in 100μl of culture medium per well were seeded into 96-well flat-bottom plates and infected by Ad-EC or Ad-GFP at MOI of 100, 50, 10 and 0 pfu/cell respectively in triplicates for 72 h, then 20 μl of 5 mg/ml MTT were added into each well and incubated for 4 h, and the optical absorbance at 570 nm was measured by a microplate reader.

### Apoptosis analysis

Apoptosis of cells were analyzed by flow cytometry. 4 × 10^5^ HEP-2 cells in 500 μl of culture medium per well were seeded into six-well flat-bottom plates, treated with Ad-EC and Ad -GFP at a MOI of 50 respectively. After incubation for 72 h, cells were harvested, stained with PE-Annexin V apoptosis detection kit (BD Biosciences Pharmingen, San Diego, CA, USA), and subjected to flow cytometry analysis.

### The xCELLigence proliferation assays

The xCELLigence Real-Time Cell Analysis (RTCA) DP instrument is an electrical impedance-based system that allows for the measurement of real-time cell proliferation [20]. XCELLigence instrumentation and protocols for the measurement of real-time proliferation were provided by Roche Diagnostics Corporation (Basel, Switzerland). 1.5 × 10^4^ cells/well HEP-2 cells were seeded on gold microelectrodes embedded at the bottom of 16 well microplates (E-plates; Roche Diagnostics, Basel, Switzerland). Drugs were added to the culture 18 h subsequent to seeding. The impedance was recorded at 15 minutes intervals.

### In vivo experiment with tumor xenograft in nude mice

The animal experiments were approved by the Ethical Committee of the Second Affiliated Hospital of Zhejiang University School of Medicine (Hangzhou, China), and carried out in accordance with the National Institutes of Health guide for the care and use of Laboratory animals. A total of 30 male mice were subcutaneously injected with 0.2 ml cell suspension of HEP-2 cells (1 × 10^7^ cells/ml) in the right dorsal scapula region. The size of tumors was measured with calipers every 3 days. When the tumor had reached the size of 50–100 mm^3^, animals were randomly divided into 6 groups and treatment of intratumoral injection was initiated as follows: group 1 received Ad-EC (1 × 10^8^ pfu/100 μl), group 2 received CDDP (2 mg/kg), group 3 received Ad-EC combined with CDDP, group 4 received Cetuximab (166μg), group 5 received Ad-GFP (1 × 10^8^ pfu/100 μl) and group 6 received PBS as a control; all administrations were given every 3 days for a total of ten doses. Tumor sizes were recorded blindly without the knowledge of the treatment groups. The volume of tumor was calculated using the simplified formula for a rotational ellipsoid V = (ab^2^)/2 (a: major axis in mm; b: minor axis in mm). Mice were euthanized by cervical dislocation at the 3^rd^ day after the final dose. The endpoint used in this animal experiment was either ten doses or the tumor xenograft size reached 2000 mm^3^.

### Measurement of tumor metabolic activity by MicroPET

MicroPET is a tomography imaging device specially used for *in vivo* experimental research on small animals. By injecting the imaging agent ^18^F-fludeoxyglucose (FDG) *in vivo*, microPET can detect the level of glucose metabolism in tumor tissue, to analyze the inhibition of metabolic activity in tumor, along with morphology evaluation. After the last administration, one mouse from each of the above groups was randomly selected and fasted for 12 h before microPET scanning. A dose of 150±20mCi ^18^F-FDG was injected into nude mice via tail vein. The nude mice were allowed to move freely for about 30 minutes before anesthetized with 2% isoflurane inhalation, then fixed to microPET examination bed and kept warm during a 10 minutes scanning using microPET R4 scanner (Siemens Medical Solutions, Malvern, PA, USA). Images were obtained after data reconstruction.

### Statistical analysis

All statistical analyses were performed with SPSS statistical software version 22 for Windows (IBM Corp., Armonk, New York, USA). The data were expressed as mean ± standard deviation (SD), and one-way analysis of variance was used to compare the values of different groups. A value of *P <* 0.05 was considered as significant.

## Results

### 1. Recombinant adenovirus Ad-EC was successfully obtained

**Fig 2:**
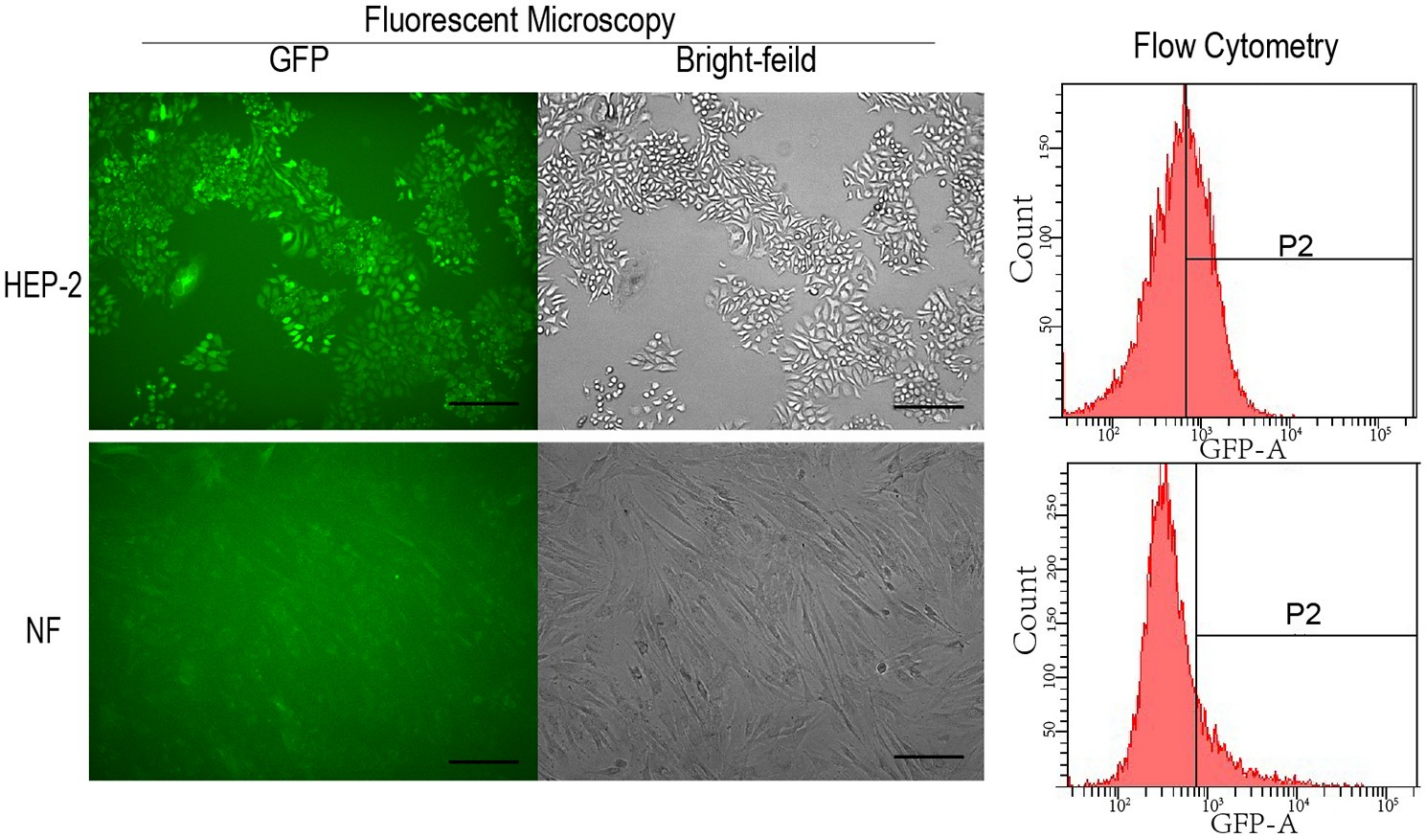
GFP expression in HEP-2 and NF cell lines after Ad- GFP infection. HEP-2 and NF cells were infected with Ad-GFP at a MOI of 50 for 72 h. GFP expression was visualized by fluorescent microscopy and quantified by flow cytometry. Bar = 200μm.

**Fig 3:**
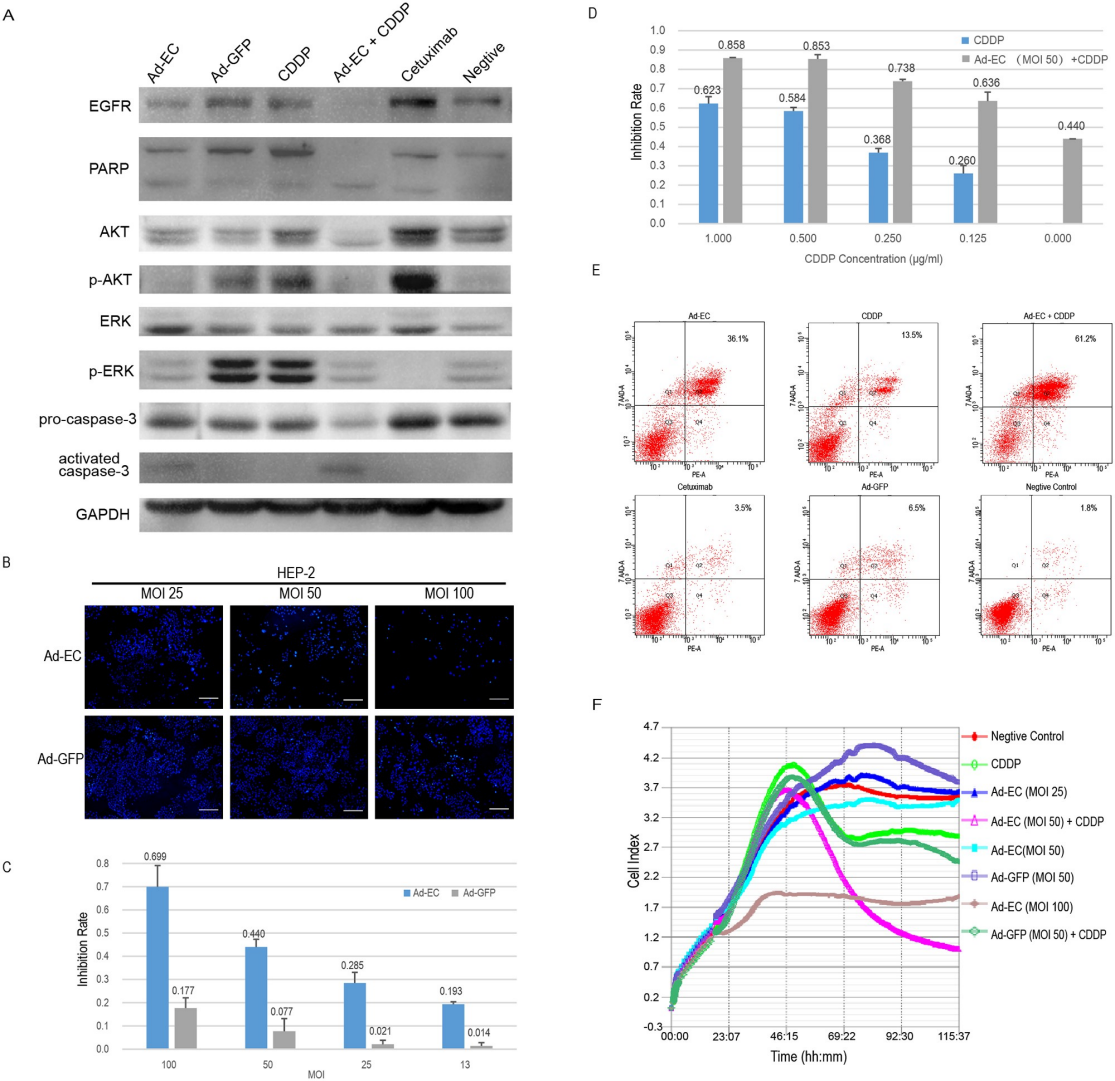
Efficient inhibition of HEP-2 cells *in vitro* by Ad-EC via inducing apoptosis. (A) HEP-2 cells were treated as follow: Ad-EC (MOI 50), CDDP (0.25μg/ml), Ad-EC (MOI 50) combined with CDDP(0.25μg/ml), Cetuximab(500μg/ml), Ad-GFP (MOI 50) or PBS. After incubating for 72 h, cells were harvested to detect the expression of following protein by Western blotting: EGFR, PARP, AKT, phosphorylated AKT, ERK, phosphorylated ERK, pro-caspase-3, activated caspase-3, and GAPDH as a control. (B) 4′6-diamidino-2-phenylindole (DAPI) staining was performed in HEP-2 cells after a 72 h infection of Ad-EC and Ad-GFP (MOI 25, 50, and 100). Bar = 200μm. (C) HEP-2 cells were infected with Ad-EC and Ad-GFP (MOI 13, 25, 50, and 100) for 72 h and assayed by MTT assay. (D) HEP-2 cells were treated with Ad-EC (MOI 50) in combination with different concentrations of CDDP (0.125. 0.25. 0.5 and 1μg/ml) for 72 h and assayed by MTT. (E) HEP-2 cells were treated in the same way as in Western blot. Apoptosis was analyzed using Annexin V/7-aminoactinomycin D (7-AAD) double-staining followed by flow cytometry after treatments. The upper right quadrant of the histograms indicated late apoptosis cells. The rates of late apoptosis cells were labeled in each group. (F) The xCELLigence proliferation assay recorded the real-time HEP-2 cell growth during the 96 h exposure of Ad-EC (MOI 25, 50, and 100), Ad-GFP (MOI 50), CDDP (0.25μg/ml), Ad-EC (MOI 50) combined with CDDP (0.25μg/ml), Ad-GFP (MOI 50) combined with CDDP (0.25μg/ml), or PBS. The cell index (CI) value is proportional to the number of living wall-attached cells before cell overgrowth.

The shuttle plasmid for recombinant adenovirus Ad-EC was successfully constructed (see S1 Fig). Ad-GFP has been previously generated by Chen et.al. [14]. After packaging, amplification, purification and concentration, the titers for Ad-EC and Ad-GFP were 6.63 × 10^9^ pfu/ml and 6.30 × 10^10^ pfu/ml respectively. Ad-GFP was validated by fluorescence microscopy and flow cytometry on GFP expression. Strong green fluorescent signals were seen in HEP-2 cells, but much weaker in NF cells 72 h after infected with Ad-GFP at a MOI of 50 (Fig 2, left), which indicated the ability of recombinant adenovirus to transduce HEP-2 cells as well as ability of SLPI promoter to drive GFP expression in these cells. Flow cytometry confirmed more GFP positive cells in HEP-2 cells than that in NF cells, with 45% in HEP-2 cells and 12% in NF cells respectively (Fig 2, right). Ad-EC was validated by Western blotting, which showed that when compared to that in Ad-GFP and negative control groups, the level of protein of 170 kd EGFR was reduced in Ad-EC group due to the expression of artificial microRNA targeting EGFR, and the activated caspase-3 was increased in Ad-EC group due to the expression of revCASP3 (Fig 3A).

### 2. Ad-EC efficiently inhibited HEP-2 cells via inducing apoptosis *in vitro*

After validation of the recombinant viruses, MTT assay was performed to evaluate the inhibition effect of Ad-EC on HEP-2 cells *in vitro*, which showed that Ad-EC had potent inhibition on HEP-2 cells in a dose-dependent manner (Fig 3B and 3C). The inhibition rate was 44.0% 72 h after Ad-EC treatment at a MOI of 50, much higher than that in Ad-GFP group which was 7.7% (Fig 3C). MOI 50 was used subsequently. The inhibition was significantly enhanced when Ad-EC was used in combination with different concentrations of commonly used chemotherapeutic drug CDDP (0.125. 0.25. 0.5 and 1μg/ml) (Fig 3D).

We also assessed the effect of Cetuximab on HEP-2 cells. The inhibition rate was only 3.9% at a concentration even as high as 500μg/ml (see S1 Table) which is much higher than the plasma concentration of clinical administration dose, indicating that HEP-2 cells are resistant to Cetuximab.

To evaluate the apoptosis-inducing potential of Ad-EC on HEP-2 cells, we quantitatively analyzed cell apoptosis after Ad-EC treatment by flow cytometry (Fig 3E). The V-PE and 7-AAD duel-positive cluster represents the late stage apoptotic cells. 72 h after Ad-EC treatment, the rate of late apoptosis was 36.1%, significantly higher than 6.5% of Ad-GFP-treated group (*P* < 0.001). In Cetuximab (500μg/ml) and CDDP (0.25μg/ml) groups, late apoptosis rate was 3.5% and 13.5%, respectively. As to the combination group (Ad-EC MOI 50 plus CDDP 0.25μg/ml), late apoptosis rate increased to 61.2%, significantly higher than each monotherapy group (*P <* 0.001), suggesting enhanced effect.

The xCELLigence system was used to measure the real-time cytotoxic response of HEP-2 cells. The cell index (CI) value reflects the impedance changes formed by the electrode at the bottom of the petri dish due to cell adherence. CI value is proportional to the number of living wall-attached cells before cell overgrowth. As shown in Fig 3F, Ad-EC (MOI 50) treatment suppressed cell growth (CI = 3.4, 72 h) when compared to negative control (CI = 3.7, 72h), and the suppression was much more significant when compared to Ad-GFP (MOI 50) (CI = 4.2, 72h). CDDP treatment (0.25μg/ml) inhibited HEP-2 cell growth after 72h (CI = 3.0, 72h), and CDDP combination with Ad-EC (MOI 50) markedly enhanced the inhibition (CI = 1.3, 72h), whereas CDDP combination with Ad-GFP had no such effect (CI = 2.8, 72h), also suggesting enhanced effect for the combination of CDDP and Ad-EC. The inhibition effect of Ad-EC on HEP-2 cells was dose-dependent (CI = 3.7 for MOI 25, CI = 3.4 for MOI 50, CI = 1.8 for MOI 100, 72h).

The recombinant adenovirus Ad-EC inhibits HEP-2 cells via both downregulation of EGFR by expressing specific targeting artificial microRNA and inducing apoptosis by expressing constitutional active reCASP3. As shown in Fig 3A, EGFR expression in Ad-EC treated HEP-2 cells was downregulated as compared to that in Ad-GFP treated HEP-2 cells, and the downregulation was more significant in Ad-EC+CDDP treated HEP-2 cells, leading to decreased p-AKT and p-ERK, two major downstream signaling pathways responsible for cellular growth and proliferation. Cleavage of PARP and activated caspase-3 were more apparent in Ad-EC treated or Ad-EC+CDDP treated HEP-2 cells, as compared to that in Ad-GFP treated HEP-2 cells, which is consistent with the results of flow cytometric analysis of apoptosis. In contrast, compared to negative control, Cetuximab treatment could not lead to the downregulation of EGFR expression in HEP-2 cells, nor cleavage of caspase-3 activation or PARP cleavage, which is also consistent with Cetuximab-resistant property of HEP-2 cells as assessed in above work. One interesting phenomenon is that Cetuximab treatment significantly inhibited phosphorylation of ERK meanwhile markedly stimulated phosphorylation of AKT, and the distinct responses of the two important signaling pathways might have consequences on HEP-2 cells’ resistance to Cetuximab.

### 3. Ad-EC efficiently inhibited tumor growth on nude mice HEP-2 xenograft

**Fig 4:**
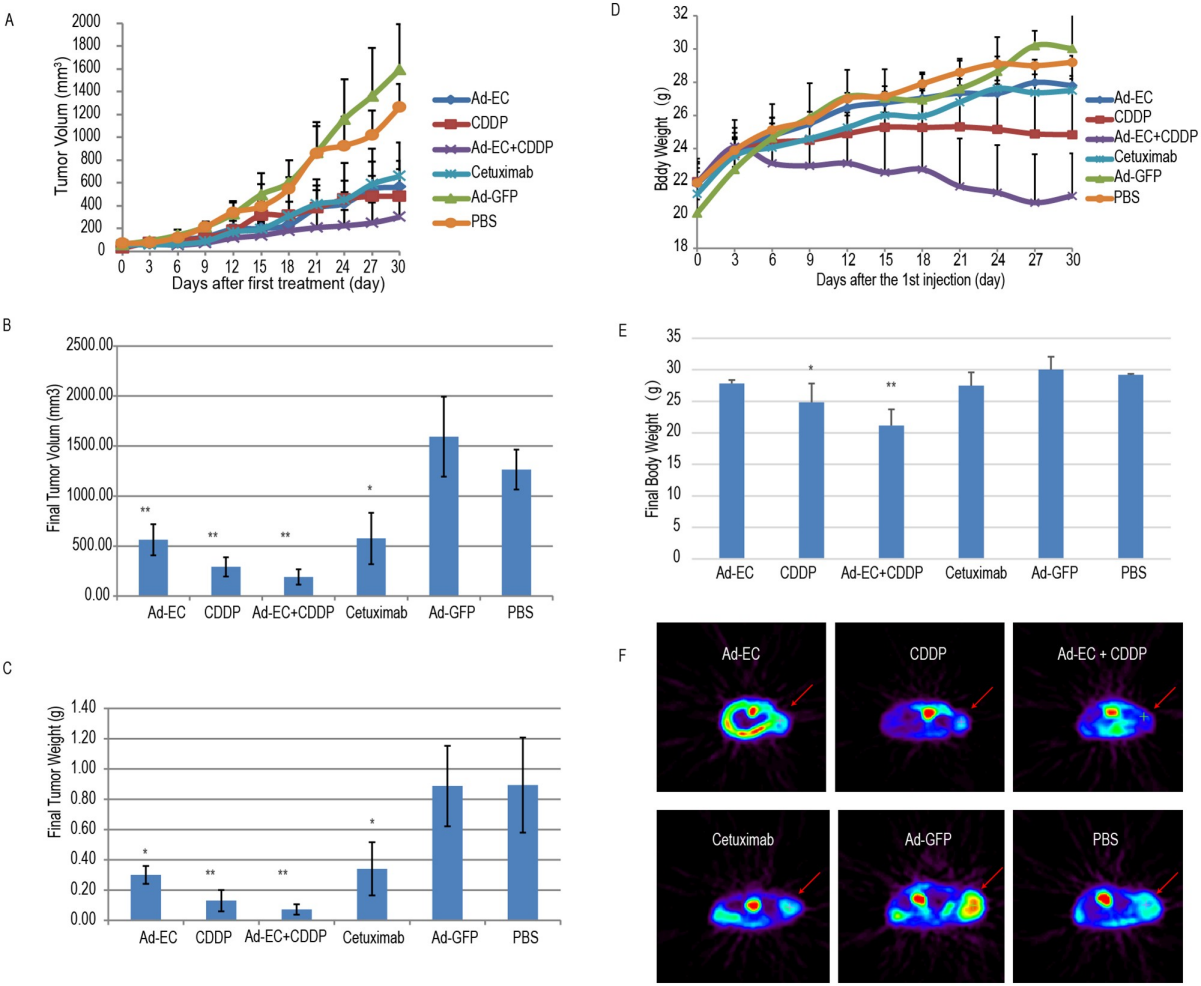
Efficient inhibition of HEP-2 xenografts in nude mice by Ad-EC. Six groups of mice received one of the following treatments by intratumoral injection every 3 days: Ad-EC, CDDP, Ad-EC combined with CDDP, Cetuximab, Ad-GFP, and PBS. Tumor sizes (A) and body weight (D) were recorded every 3 days. Three days after the 10^th^ injection, the final tumor size (B), tumor weight (C) and body weight (E) were recorded. *P < 0.05; ** *P <* 0.01. (F) MicroPET CT scanning was taken to measure tumor metabolic activity.

In this study all mice received 10 doses of different administrations, none of the xenograft reached 2000 mm^3^ size endpoint at the time of the last measurement before euthanasia and none of the mouse died before euthanasia. Ad-EC and Ad-GFP were given by intra-tumor injection at a 1 × 10^8^ pfu/100 μl/mouse once every three days. As shown in Fig 4, compared with Ad-GFP treated and PBS control groups, the tumor xenograft growth rate was significantly inhibited with smaller final tumor volume and lower tumor weight in Ad-EC treated group. (*P* < 0.05) (Fig 4A~4C) at the 30^th^ day after administration, while the body weight and daily behavior of nude mice were similar to the control groups. The tumor growth rate, final tumor volume and tumor weight of the CDDP group were significantly lower than that of the control groups (*P* < 0.05) (Fig 4A~4C), but the body weight of the nude mice was decreased significantly compared with that of the control groups (*P* < 0.05) (Fig 4D~4E) which suggested severe side effect. The tumor inhibition effect was markedly enhanced by the combination of Ad-EC and CDDP treatment, but the side effect was also more pronounced than that of the CDDP treatment group (*P* < 0.05) (Fig 4A~4E), as shown by the body weight.

As expected, the tumor volume and FDG uptake signal were significantly decreased in the Ad-EC, CDDP, Ad-EC+CDDP and Cetuximab treated groups at 30^th^ days compared with the PBS and Ad-GFP treated control groups; especially in the Ad-EC+CDDP combination group, tumor tissue FDG uptake was as low as normal tissues, indicating that tumor metabolism was significantly inhibited (Fig 4F).

We observed *in vivo* tumor inhibition in Cetuximab treated group in this experiment (*P <* 0.05, compared with PBS group), which was unexpected and different from the *in vitro* results.

## Discussion

EGFR has long been a popular target in cancer therapy, as the over-expression and/or over-activation of EGFR contributes to the malignant growth, metastasis and poor prognosis of various cancers [2, 21, 22]. However, all patients with metastatic lung, colorectal, pancreatic or head and neck cancers who initially benefit from EGFR-targeted therapies eventually develop resistance [1].

In this study, we found that EGFR over-expressed cell line HEP-2 was resistant to Cetuximab *in vitro*, which is consistent to reports [23]. When compared with negative control group, markedly increased EGFR expression was observed after Cetuximab treatment, probably due to the compensating response to downregulation of EGFR pathway [8]. Although ERK phosphorylation was suppressed, AKT activation was significantly enhanced which was considered to be one of the Cetuximab resistance mechanisms [8, 24].

To overcome the drug-resistance challenge, we employed RNA interference to inhibit EGFR synthesis at mRNA level. Artificial microRNA (second generation shRNA) preserves the specific sequence targeting a gene present in shRNA, just as siRNA and first-generation shRNA. But it has the advantage that its transcription can be initiated by most promoters in mammals (like SLPI promoters used in this study), owing to its microRNA frame [25]. Another advantage of this strategy is that resistance to other therapeutics caused by EGFR mutation can be avoided by targeting non-mutational conserved sequences. The designed microRNA can form functional short single-stranded RNA after intracellular metabolism, which matches the junction between the untranslated region (UTR) of mRNA and the first codon, leading to mRNA degradation and down-regulation of EGFR protein synthesis. The Western blotting in this study and our previous study [10] have confirmed that EGFR expression in HEP-2 cells was successfully decreased by this microRNA.

The levels of phosphorylated ERK and AKT in the Ad-EC group were effectively inhibited, confirming that EGFR signaling pathway was blocked more efficiently by microRNA and could overcome the resistance of HEP-2 cells to monoclonal antibody Cetuximab. Thus, inhibiting EGFR expression at mRNA level can avoid the reported resistance mechanisms such as EGFR overexpression, EGFR degradation disorders, and bypass activation of EGFR downstream molecules (especially AKT pathway).

Cetuximab treatment exhibited somewhat anti-tumor effect in HEP-2 xenografts in nude mice in this study, though not as effective as Ad-EC treatment or CDDP treatment. As i*n-vitro* experiments suggested that blocking the extracellular domain of EGFR by Cetuximab is not sufficient to suppress tumor growth, Cetuximab might elicit antibody-dependent cell-mediated cytotoxicity (ADCC) or complement dependent cytotoxicity (CDC) *in vivo* [24], the latter may led to the inhibition of tumor xenograft in T cell-defective nude mice observed in this study.

Caspase-3 plays a key role in apoptosis. The recombinant caspase-3 revCASP3 with its large and small subunits rearranged is constitutively active and able to induce cell apoptosis directly without the need for activation by the upstream caspases [13]. Our previous work has demonstrated that the expression of revCASP3 under the control of tumor specific promotor SLPI could effectively activate endogenous caspase-3 and subsequent apoptosis of HEP-2 cells [14]. In current study, we adopted the strategy to combine revCASP3 and EGFR artificial microRNA. As expected, EGFR was significantly downregulated along with remarkable increase of activated caspase-3 and cleaved PARP levels in Ad-EC treated HEP-2 cells when compared with those in Ad-GFP treated HEP-2 cells, further confirmed by flow cytometric analysis data for apoptosis *in vitro* and tumor suppression on HEP-2 xenograft in nude mice *in vivo*. Notably, Ad-EC treatment did not suppress weight gain of mice during a 30 days long treatment, indicating this recombinant adenovirus is effective and well tolerated.

CDDP (cisplatin) is a first line option of systemic therapy for advanced cancers (e.g. cervical cancer, head & neck cancers). CDDP is a heavy metal complex that cross-links with DNA, inhibiting DNA replication and inducing apoptosis [26]. Significantly enhanced antitumor effect was achieved when CDDP was combined with Ad-EC both i*n vitro* and *in vivo*. Moreover, normal level of FDG uptake activity in microPET scanning was observed on the 30^th^ medication day in CDDP + Ad-EC treated HEP-2 xenograft harbored mouse. Interestingly, although CDDP alone did neither downregulate EGFR nor activate pro-caspase-3, CDDP + Ad-EC showed profound EGFR downregulation and caspase-3 activation (Fig 3A). Ad-EC administration showed comparable therapeutic effect to CDDP administration while no any systemic adverse effect such as diarrhea or on weight gain, much better than CDDP (Fig 4). However more significant adverse effect was observed when Ad-EC and CDDP were combined, as indicated by the weight gain inhibition, though the combination led to better tumor suppression. The reason remain unknown and further investigation is needed in the future to explore ideal therapeutic options with enhanced efficacy and less side effects. Replication-deficient recombinant adenovirus is one of the most efficient vectors for gene delivery and expression. We used adenovirus as a gene delivery vector because of its properties including the safety for non-integration into genome of host cells, easy manipulation in vector packaging and high titer in virus preparation (10^12^−10^13^ pfu/ml).

Controlled gene expression by tissue-specific promoters is one of the most effective ways for targeted gene therapy. The tumor-specific promoters can restrict expression of therapeutic genes in tumor cells and therefore minimize the risk of nonspecific side effects on normal tissues. In previous study, we confirmed that SLPI promoter had 37-fold higher transcription activity in HEP-2 cells than human umbilical vein endothelial cells using luciferase assay [14]. In this study, we found that the expression level of SLPI promoter-controlled GFP in HEP-2 is much higher than that in normal fibroblast 72 h after Ad-GFP infection, which might be due to differential infection or transcription, this further confirmed the tumor-specificity of this adenoviral gene therapy strategy.

In summary, the recombinant adenovirus Ad-SLPI-EGFRamiR-SLPI-revCASP3, armed with EGFR targeted artificial microRNA and recombinant caspase-3 under the control of tumor specific promoter SLPI, was successfully constructed. The results revealed good tumor-targeting effect of the recombinant adenovirus Ad-EC with the tumor specific promoter modulated expression of revCASP3 and artificial microRNA targeting EGFR. and the dual-target gene therapy exhibits a satisfactory tumor inhibitory effect compared to modalities currently available to clinics, CDDP and Cetuximab.

## Supporting information

S1 Fig Characterization of recombinant adenovirus Ad-EC. (A) Restriction endonuclease digestion of pDC312-SLPI-miEGFR-pA-SLPI-revCasp3-TAG-pA by *Eco*RI. M.1 Kb Plus DNA Marker; 1–3. Digestion by *Eco*RI showing 4252bp, 1888bp and 1239bp bands. (B) Characterization of recombinant adenovirus of Ad-EC by PCR. M. DL2000 DNA Ladder; 1. Amplified product of the supernatant for Ad-EC packaging (879bp).
(TIF)

S1 Table Cytotoxicity of Cetuximab to HEP-2 cells by MTT assay. (DOCX)

S1 Raw images (PDF)
