# Supplementary figures and images for: Effective inhibition of cancer cells by recombinant adenovirus expressing EGFR-targeting artificial microRNA and reversed-caspase-3
Source: PLoS One. 2020 Aug 3;15(8):e0237098. doi: 10.1371/journal.pone.0237098 (PMC7398494; doi:10.1371/journal.pone.0237098)

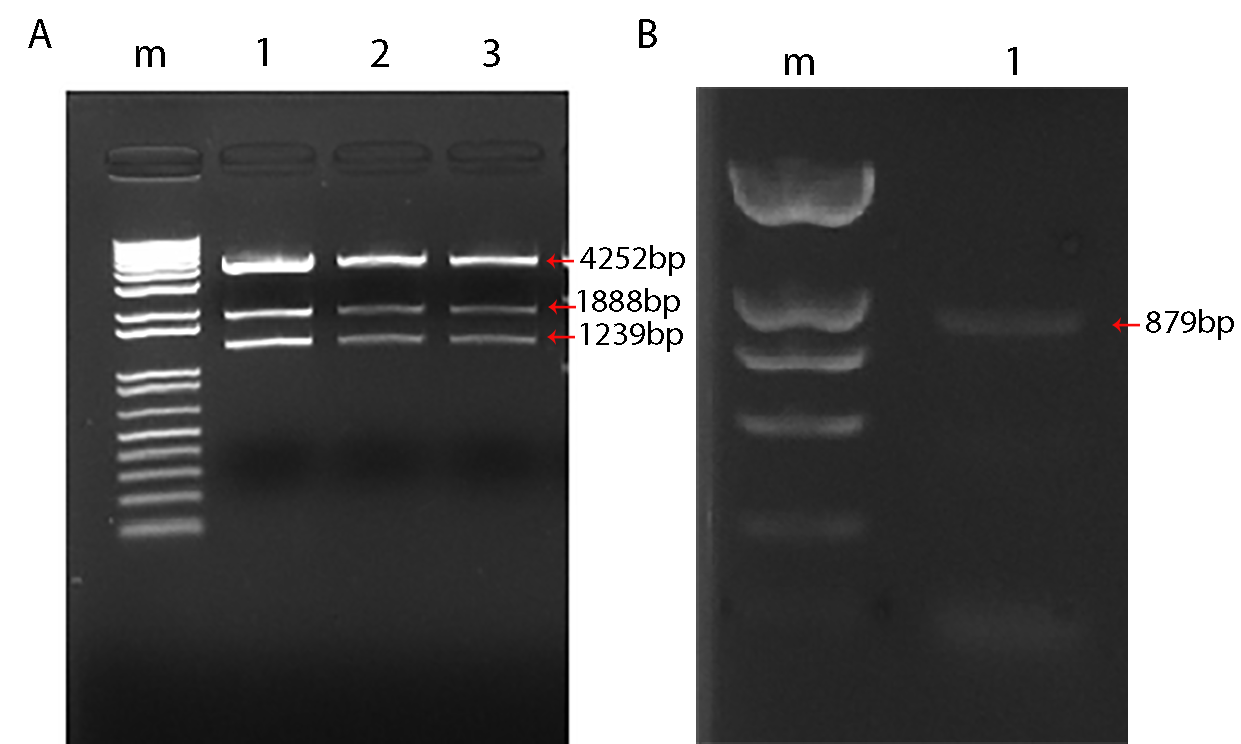

Supplement: S1 Fig — (A) Restriction endonuclease digestion of pDC312-SLPI-miEGFR-pA-SLPI-revCasp3-TAG-pA by EcoRI. M.1 Kb Plus DNA Marker; 1–3. Digestion by EcoRI showing 4252bp, 1888bp and 1239bp bands. (B) Characterization of recombinant adenovirus of Ad-EC by PCR. M. DL2000 DNA Ladder; 1. Amplified product of the supernatant for Ad-EC packaging (879bp). (TIF) [file pone.0237098.s001.tif]

The original gel of S1 Fig. A.

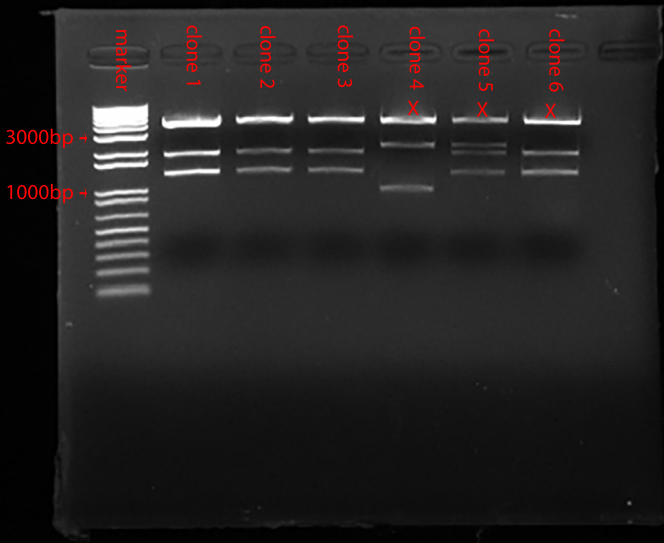

The original gel of S1 Fig. B.

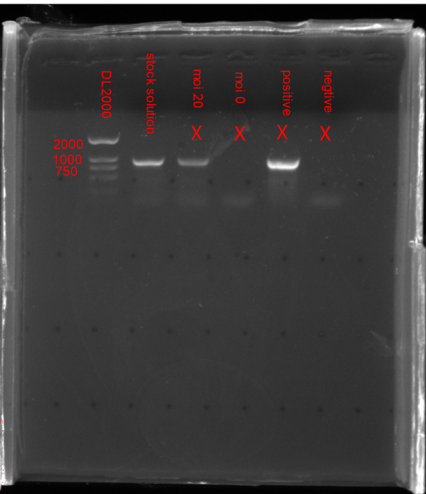

The original blot image of Fig.3 F.

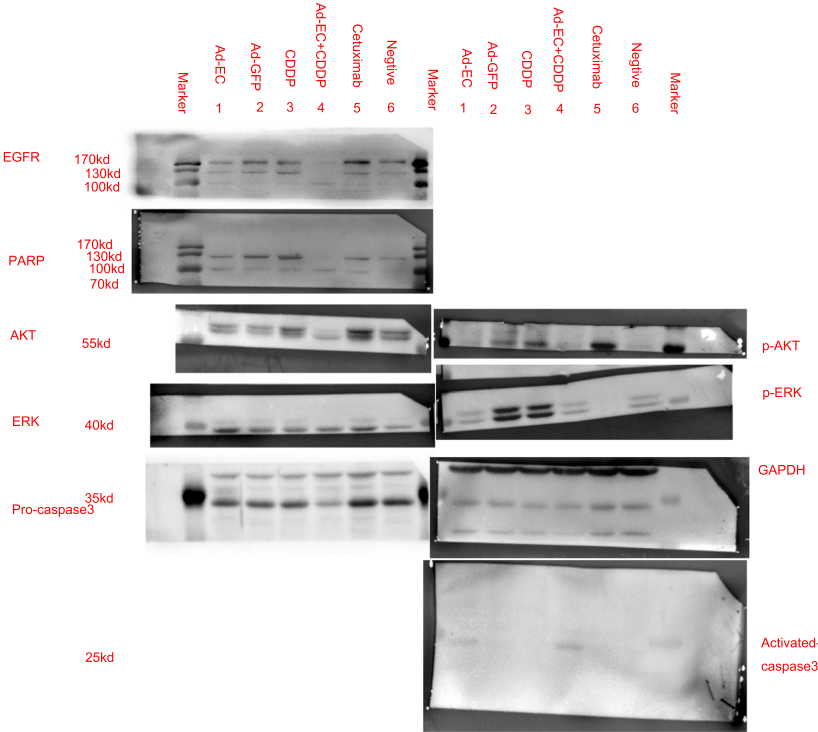

Supplement: S1 Raw images — (PDF) [file pone.0237098.s003.pdf]
