# Supplementary material for: Effective inhibition of cancer cells by recombinant adenovirus expressing EGFR-targeting artificial microRNA and reversed-caspase-3
Source: PLoS One. 2020 Aug 3;15(8):e0237098. doi: 10.1371/journal.pone.0237098 (PMC7398494; doi:10.1371/journal.pone.0237098)
Supplement: S1 Table — (DOCX) [file pone.0237098.s002.docx]

S1_Table. Cytotoxicity of Cetuximab to HEP-2 cells by MTT assay

Cetuximab (μg/ml) OD_570nm_ (Mean±SD) Inhibition Rate

1000 0.29±0.05 34.4%

500 0.43±0.01 3.9%

250 0.42±0.02 5.2%

125 0.44±0.01 1.7%

0 0.45±0.01
